# Thyroid Hormones and Peripheral Nerve Regeneration

**DOI:** 10.1155/2013/648395

**Published:** 2013-04-02

**Authors:** Ioannis D. Papakostas, George A. Macheras

**Affiliations:** Department of Pharmacology, University of Athens, 75 Mikras Asias Avenue, Goudi, 11527 Athens, Greece

## Abstract

Peripheral nerve regeneration is a unique process in which cellular rather than tissue response is involved. Depending on the extent and proximity of the lesion and the age and type of the neuronal soma, the cell body may either initiate a reparative response or may die. Microsurgical intervention may alter the prognosis after a peripheral nerve injury but to a certain extent. By altering the biochemical microenvironment of the neuron, we can increase the proportion of neurons that survive the injury and initiate the reparative response. Thyroid hormone critically regulates tissue growth and differentiation and plays a crucial role during organ development. Furthermore, recent research has provided new insight into thyroid hormone cellular action. Thyroid hormone regulates stress response intracellular signaling and targets molecules important for cytoskeletal stability and cell integrity. Changes in thyroid hormone signaling occur in nerve and other tissues, with important physiological consequences. The interest in thyroid hormone in the context of nerve regeneration has recently been revived.

## 1. Introduction

Thyroid hormones are essential for the development and maturation of the central nervous system. There is a period in brain development in which the euthyroid status is a prerequisite for normal development. Thyroid hormones promote morphogenesis and function in various areas of the brain. The molecular basis of the thyroid dependent brain development is not yet clarified [1]. Among other processes, defects in myelination, delays in the development of the dendritic tree, decreased number of glial cells, and axo-dendritic synapses have all been described in hypothyroid newborn rats.

Peripheral nerve injuries are very common in the clinical setting [2]. These injuries are very debilitating, commonly affecting the younger more productive portion of the population. Microsurgical techniques to restore nerve continuity have reached their limits, and further surgical advancements in the field of peripheral nerve surgery are unlikely to improve the prognosis. A different approach based on the cellular and molecular aspects of regeneration should be followed [3]. This approach is justified by the fact that peripheral nerve injuries are in reality cellular, rather than tissue injuries. It is the long axon of the neuron that has been injured. In response, the cell body adapts in an effort to restore the cellular volume and function.

Before adaptation the neuron should first survive from the index event. Apoptosis can be the fate of a large proportion of cell bodies. There are many interplaying factors that define the future of the neuron. These factors can be modulated both by surgical intervention and by altering biologically the microenvironment of the cell. The second important process is cell adaptation. The neuron should change its metabolic state promoting cell regeneration. These are important steps in the process of regeneration with cellular and molecular parameters influencing the final result.

## 2. Nerve Regeneration

The process of nerve regeneration is actually a cellular response to injury. The nerve cell after the traumatic event loses a portion of its intracellular volume and initiates an effort to restore its shape and function. Nerve regeneration is only one aspect of the problem. Appropriate target reinnervation and central nervous system adaptation are other equally important processes in restoration of function.

Many studies have been undertaken to more efficiently define the biochemical and cellular processes of nerve regeneration. A large proportion of these studies use the chamber model. With this model a conduit is used to bridge the interstump gap. The proximal and distal nerve stumps are introduced and secured at the ends of the conduit material, leaving a predefined interstump distance. This conduit can be of organic or synthetic origin. Veins, mesothelial tissues, collagen sheets, biosynthetic materials, biodegradable materials, and silicone have all been used [4].

Silicone tubes can be used very efficiently to bridge the interstump gap. Silicone as a material has many important advantages as follows:

- availability in various sizes and diameters,
- ability to be sterilized,
- cheapness,
- sufficiently rigid to withstand compression,
- easily handled surgically for suture penetration,
- biologically inertness,
- not inducing inflammation or resorption and thus not affecting the process of regeneration.

The introduction of the chamber model was made on the assumption that eliminating the influence of the external environment would be beneficial for the process of regeneration. Molecules and cells derived from the distal and proximal nerve stumps are concentrated inside the chamber undisturbed from exogenous factors. The results of those pilot studies were very encouraging as gaps 1 cm in length were successfully bridged in small animal models (rats) [5, 6]. 

An advantage of a closed chamber model is the ability offered to alter the biochemical or cellular microenvironment within the gap. With this model many studies have been conducted examining the role of various factors in regeneration. The role of neurotrophic and neurite promoting factors has been investigated. The process of regeneration itself can also be studied efficiently in a timely manner, as the contents inside the chamber can be examined at various intervals from implantation. Biochemical, cellular, and histological methods can be used to delineate the various stages and processes of regeneration [7].

The implications of thyroid hormones in peripheral nervous system have not been studied adequately. Moreover earlier studies showed conflicting results [8, 9]. These studies failed to detect any change in the rate of growth of sensory axons with T3 treatment, after a sciatic nerve crush. These earlier studies on peripheral nerve regeneration, in order to achieve higher concentrations of the hormone at the site of regeneration, used large quantities of T3 intraperitoneally. One possible explanation of these discouraging results is that these large quantities might had a detrimental role, acting systematically, masking any potential benefit. The introduction of the silicone chamber model in the study of peripheral nerve regeneration facilitated the application of small quantities of the hormone in a confined space thus eliminating adverse systemic effects. 

## 3. The Nuclear Receptors of Thyroid Hormones

Thyroid hormones exert their function either through their extranuclear (nongenomic) or through their genomic action on the cell. The extranuclear action is mediated through the direct effect on membrane ion channels and pumps [10]. Their genomic action requires specific nuclear receptors (thyroid hormone receptors, TRs). These receptors after activation regulate the transcription of various genes. TRs have been classified in two families according to the genes (TR*α*, TR*β*) that encode these receptors. Different processing of the TR*α* gene transcript results in the formation of three isoforms (splice variants): TR*α*1, TR*α*2, and TR*α*2v. TR*β* gene isoforms include TR*β*1 and TR*β*2. Of all these isoforms, only TR*α*1, TR*β*1, and TR*β*2 promote specific gene expression after their activation. TR*α*2 and TR*α*2v activation does not lead to specific gene expression [11].

The localization of TRs in the peripheral nervous system is of great importance in the understanding of the mode of action of T3 in nerve development and regeneration in rats. In peripheral nerves, TRs are expressed in Schwann cells during a defined period, from the third week of embryonic life through the second postnatal week. This period is characterized by active proliferation of Schwann cells. After this period Schwann cells fail to express TRs. The first Schwann cells to lose the expression of TRs are those in close proximity with the neuronal body. This elimination of TRs expression is concomitant with the process of myelination and follows the temporospatial pattern of myelination from proximal to distal [11]. Thus Schwann cells distally retain their ability to express TRs longer than the more proximally located cells. This loss of TRs expression from Schwann cells persists throughout adult life in intact peripheral nerves. After a nerve injury, the Schwann cells reexpress thyroid nuclear receptors. This expression involves mainly the distal stump of the transected nerve and follows again the same temporospatial pattern but this time in reverse. The first Schwann cells to reexpress TRs are those in close proximity with the lesion with the more peripheral cells to follow. This process is concomitant with the process of degeneration and myelin degradation in the peripheral stump from proximal to distal. The expression of TRs precedes a phase of active Schwann cell proliferation. Thus it can be inferred that the loss of contact of axons with the Schwann cells in the peripheral stump promotes TRs expression. This loss of axon-cell contact leads to major physiological changes switching Schwann cell to an embryonic state of proliferation and of expression of specific neurotrophic and neurotropic genes [11].

Sensory neurons in dorsal root ganglia express TRs throughout their life. Thus thyroid hormones play a continuous functional role in sensory neurons. Their action is not confined temporally on prenatal maturation and development. In the spinal cord, TRs expression is constant at the large motor neurons and the other neurons of the grey matter. One difference from the peripheral glial cells is that the glial cells of the central nervous system, namely, the oligodendrocytes and the astrocytes, express TRs constantly, irrespective of the axonal contact or the presence of myelin [11]. 

Schwann cells express predominantly the TR*α*1 splice variant and to a lesser degree the TR*α*2 and TR*β*1. TR*α*1 is the predominant type during development and during regeneration. The predominant TRs in sensory neurons of the dorsal root ganglia are TR*β*1 and TR*α*2. From the above, it can be inferred that the postactivation cascade involves different mechanisms between Schwann cells and sensory neurons. Furthermore it has been found that throughout the nervous system the population of nonfunctional TRs isoforms is very large. This population may play a role in the regulation of the circulating levels of thyroid hormones [11].

## 4. Mechanisms of Action of Thyroid Hormones

A proportion of sensory neurons in the dorsal root ganglia will undergo a process of apoptosis after a traumatic event as described previously. T3 is likely to have a direct effect on these neurons lessening the degree of apoptosis and rescuing a significant number of sensory neurons [12]. By rescuing sensory neurons, T3 increases the number of new axons that enter the distal stump. This action is a direct effect and is not mediated through the action of T3 on peripheral glial cells. There are the results of two different types of experiments in support for the above. 

The first group of experiments uses the silicone chamber model to investigate the fate of the application of 125 I—labeled T3 in the chamber. It has been documented that the neuronal bodies in the dorsal root ganglia show labeling after injection of the labeled T3 in the silicone chamber. It has been proposed that T3 is retrogradely transported through the proximal stump to the neuronal body. In the neuronal body, T3 binds to the nuclear receptors and exert its action rescuing the neuron from death [11].

The second group of experiments uses cell cultures prepared from dorsal root ganglia (DRG) of rat embryos. Three types of cultures were prepared: (a) mixed dissociated DRG cultures, with neurons and nonneuronal cells including Schwann cells, (b) primarily neuronal cell culture with diminished number of nonneuronal cells, and (c) explant DRG culture where the physical relationship between neuronal and nonneuronal cells was preserved. The addition of T3 at physiological concentration resulted in the stimulation of neuron survival to the same extent for both mixed and neuron enriched cultures. Thus it can be inferred that T3 acts directly on neurons promoting their survival [11]. This effect is not mediated by neurotrophic factors secreted by nonneuronal cells after their activation by T3. This action is probably mediated through TR*β*1 as this is the predominant receptor in sensory neurons [11].

T3 is also promoting the outgrowth of new axons from sensory neurons. This effect was observed in explant DRG cultures where the relationship of neuronal and nonneuronal cells was largely preserved. This increased outgrowth was not observed in neuron enriched cultures or when the proliferation of nonneuronal cells was inhibited by antimitotic agents [11]. Thus it can be inferred that the promotion of neurite outgrowth is mediated by the action of T3 on nonneuronal cells. Nonneuronal cells under the influence of T3 may secrete neurotrophic factors that promote neurite outgrowth [11]. These factors may include NGF (nerve growth factor), NT-3 (neurotrophin 3), and possibly others.

T3 acts on Schwann cells through TR*α*1 and on neuronal bodies through TR*β*1. Thus it can be inferred that T3 uses multiple different processes to promote neuronal survival and neurite outgrowth. The molecular basis of the cascade of events after the binding of T3 on its specific receptors is not yet clarified but the end result is the activation of specific target genes [13, 14].

Formation and transport of cytoskeletal proteins are another mode of possible T3 action promoting regeneration. T3 seems to enhance the anabolic activity of the neuronal body. Neurofilament subunits and tubulin isoforms levels have been found, by immunocytochemistry and western blot analysis, to increase in response to local T3 treatment. These necessary elements for neurite outgrowth have been found more rapidly in the distal segments of the regenerating nerve. T3 possibly enhances the anterograde transport of these molecules by slow axonal transport. Thus under the influence of local T3 new axons enter more rapidly and in greater numbers the distal stump [15]. 

Another implication of the increased levels of neurofilaments in the regenerating distal stump is the actual increase of the axon diameter, as there is a direct relationship between the amount of neurofilaments subunits and the radial growth of the new axon. The levels of tyrosinated and acetylated tubulin are also increased after T3 treatment. Microtubules which form the framework of the axons are primarily composed of tubulin and various microtubule-associated proteins which play an important structural and functional role. T3 through a synergistic action with NGF seems to regulate the formation, the transport, and the assembly of these proteins [15].

Perhaps one of the most striking effects in peripheral nerve regeneration of local T3 is the increase in formation of the myelin sheath of new axons. Many histological studies have documented such an improvement in myelination. Thus T3 treatment may improve the function of motor neurons with its effect on maturation and the increase in the diameter of the myelin sheath. This effect of T3 is probably a direct stimulatory effect on Schwann cells. T3 may also act by increasing the proliferation of Schwann cells as has been shown by the increased incorporation of [3H]thymidine in T3 stimulated cultures of Schwann cells [11].

Our study [16] investigated potential functional effects of thyroid hormone on peripheral nerve regeneration in rats. After complete transection of the right sciatic nerve, the gap between the stumps was bridged with a silicone tube. In the first experimental group T3 solution was used to fill the tube, while a sterile buffer solution was used in nontreated rats. Additionally, sham operation with surgical incision and mobilization of the sciatic nerve without any other intervention was performed. In a few animals, a segment of the nerve was excised and the stumps were reversed to exclude the possibility of regeneration. The process of peripheral nerve regeneration was assessed by functional indices at three, six, nine, thirteen, and seventeen weeks postoperatively [17–19]. Midstance angle, at the ankle, measured in degrees, was used as a kinematic index [20], and the withdrawal reflex (measured in grams of applied force) was used to evaluate the return of sensory function. Kinematic indexes were not different between groups A and B at all time points of the evaluation. Sensory function was significantly different in T3 treated animals compared to buffer treated control group (*x* versus *y*, *P* = 0.031) at nine weeks. Thereafter, sensory function was comparable between groups.

At molecular level, it is likely that T3 upregulates stress responsive survival intracellular signaling. In fact, phosphorylation of ERK is induced by thyroid hormone at the microenvironment of nerve regeneration [21]. Another mechanism may be through the upregulation of heat shock proteins, which can increase the rate of survival of motor [22] and sensory [23] neurons after injury and induce the process of nerve regeneration [24, 25]. However, although thyroid hormone has been shown to induce the expression and phosphorylation of heat shock protein 27 in heart tissues [26], it remains largely unknown whether similar effect can occur in nerve tissue. This issue merits further investigation.

In conclusion local T3 acts rapidly and efficiently, enhancing a wide variety of different mechanisms which promote regeneration [27]. Due to the short half-life of T3, it is probable that the concentration of the molecule inside the silicone tube rapidly decreases. Thus T3 should act rapidly in many targets which in turn produce a lasting effect to promote, through various mechanisms, peripheral nerve regeneration.
